# RDX Binds to the GABA_A_ Receptor–Convulsant Site and Blocks GABA_A_ Receptor–Mediated Currents in the Amygdala: A Mechanism for RDX-Induced Seizures

**DOI:** 10.1289/ehp.1002588

**Published:** 2010-11-10

**Authors:** Larry R. Williams, Vassiliki Aroniadou-Anderjaska, Felicia Qashu, Huckelberry Finne, Volodymyr Pidoplichko, Desmond I. Bannon, Maria F. M. Braga

**Affiliations:** 1 U.S. Army Public Health Command (Provisional), Aberdeen Proving Ground, Maryland, USA; 2 Department of Anatomy, Physiology, and Genetics; 3 Department of Psychiatry and; 4 Neuroscience Program, F. Edward Hébert School of Medicine, Uniformed Services University of the Health Sciences, Bethesda, Maryland, USA

**Keywords:** amygdala, GABA_A_ currents, GABA_A_ receptors, RDX, seizures

## Abstract

**Background:**

Hexahydro-1,3,5-trinitro-1,3,5-triazine (RDX) is a high-energy, trinitrated cyclic compound that has been used worldwide since World War II as an explosive in both military and civilian applications. RDX can be released in the environment by way of waste streams generated during the manufacture, use, and disposal of RDX-containing munitions and can leach into groundwater from unexploded munitions found on training ranges. For > 60 years, it has been known that exposure to high doses of RDX causes generalized seizures, but the mechanism has remained unknown.

**Objective:**

We investigated the mechanism by which RDX induces seizures.

**Methods and results:**

By screening the affinity of RDX for a number of neurotransmitter receptors, we found that RDX binds exclusively to the picrotoxin convulsant site of the γ-aminobutyric acid type A (GABA_A_) ionophore. Whole-cell *in vitro* recordings in the rat basolateral amygdala (BLA) showed that RDX reduces the frequency and amplitude of spontaneous GABA_A_ receptor–mediated inhibitory postsynaptic currents and the amplitude of GABA-evoked postsynaptic currents. In extracellular field recordings from the BLA, RDX induced prolonged, seizure-like neuronal discharges.

**Conclusions:**

These results suggest that binding to the GABA_A_ receptor convulsant site is the primary mechanism of seizure induction by RDX and that reduction of GABAergic inhibitory transmission in the amygdala is involved in the generation of RDX-induced seizures. Knowledge of the molecular site and the mechanism of RDX action with respect to seizure induction can guide therapeutic strategies, allow more accurate development of safe thresholds for exposures, and help prevent the development of new explosives or other munitions that could pose similar health risks.

Hexahydro-1,3,5-trinitro-1,3,5-triazine (RDX; royal demolition explosive) is a high-energy cyclic compound developed before World War II and has a history of worldwide use in explosive mixtures and formulations, such as C-4 (McLellan et al. 1992; Yinon 1990). RDX can be released into the environment by way of waste streams generated during the manufacture, use, and disposal of the pure product or RDX-containing munitions (McLellan et al. 1992). Another possible route of exposure is unexploded munitions found on training ranges, with the accompanying risk of groundwater leaching [Agency for Toxic Substances and Disease Registry (ATSDR) 2010]. The risk of inadvertent release to groundwater or soil has resulted in U.S. Environmental Protection Agency (EPA) regulations governing human oral exposure. Based on more recent studies (Crouse et al. 2006; Gust et al. 2009; Kasuske et al. 2009; Kucukardali et al. 2003), the U.S. EPA is currently reassessing RDX information available in the Integrated Risk Information System database (U.S. EPA 1993).

For > 60 years it has been known that human exposure to high doses of RDX causes headache, dizziness, vomiting, and confusion, followed by tonic-clonic seizures (Barsotti and Crofti 1949; Kasuske et al. 2009; Kucukardali et al. 2003). Although initial isolated reports were of accidental exposure during manufacture or other contamination (ATSDR 2010; U.S. EPA 1993), case reports peaked during the Vietnam War, when RDX was inadvertently ingested by soldiers using it to ignite cooking fires, or intentionally taken as an illicit intoxicant (Stone et al. 1969); such case reports still appear (Kasuske et al. 2009). Intense seizures and status epilepticus after experimental RDX exposure have also been observed in a wide range of species, from lizards and birds to nonhuman primates (Burdette et al. 1988; Crouse et al. 2006; Gust et al. 2009; Martin and Hart 1974; Quinn et al. 2009; Schneider et al. 1977; Von Oettingen et al. 1949). It has been speculated that the limbic plays a primary role in RDX-induced seizures (Burdette et al. 1988), but the cellular mechanism of seizure induction has been unknown. Here, we present evidence that RDX binds at the convulsant site of the γ-aminobutyric acid type A (GABA_A_) receptor (Benarroch 2007; Johnston 2005; Kalueff 2007), causing reduction of GABA_A_ receptor–mediated synaptic transmission and induction of epileptiform activity, as we demonstrate in the amygdala, a seizure-prone structure of the limbic system (Aroniadou-Anderjaska et al. 2007, 2008).

## Materials and Methods

### RDX

We obtained RDX from the Department of the Navy (Naval Ordnance and Security Activity, Indian Head, MD); HPLC analysis showed it to be > 99.5% pure, with the remainder being water. For animal experiments, RDX was dissolved in 1% methylcellulose and 0.2% Tween-80 and administered to rats by oral gavage at the dose of 75 mg/kg, a dose that consistently induces seizures within 30 min after administration (McCain W, personal communication). Control rats received a vehicle solution that consisted of 1% methylcellulose and 0.2% Tween-80 without RDX; vehicle was also administered by oral gavage. For *in vitro* studies, RDX was dissolved in dimethyl sulfoxide (DMSO) at a 100× concentration to minimize the percentage of DMSO in the final dilution.

### *In vivo* electrophysiological experiments

Male Sprague-Dawley rats were individually housed in an environmentally controlled room (20–23°C; 12-hr light/dark cycle, with lights on 0600 hours), with food and water available *ad libitum*. The rats weighed 150–250 g at the beginning of the experiments. All animal experiments were approved by the Institutional Animal Care and Use Committee of the U.S. Army Center for Health Maintenance and Preventive Medicine and the Uniformed Services University of the Health Sciences. The investigators adhered to the *Guide for the Care and Use of Laboratory Animals* (Institute of Laboratory Animal Resources 1996), which requires humane treatment of all animals, with regard for alleviation of suffering.

Rats were anesthetized with a combination of ketamine [80 mg/kg, intraperitoneally (IP)] and xylazine (10 mg/kg, IP), and cortical stainless steel electrodes (Plastics One, Roanoke, VA) were stereotaxically implanted over the left and right frontal cortex (2.0 mm posterior and 2.5 mm lateral to bregma) and left and right parietal cortex (5.5 mm posterior and 2.5 mm lateral to bregma), with a reference electrode implanted over the cerebellum (1.5 mm posterior from lambda). After 1 week of recovery, rats were placed in the electroencephalogram (EEG) chamber and connected to the EEG system (Stellate, Montreal, Canada). Video-EEG recordings of baseline activity in the freely moving rats began at least 10 min before oral dosing of RDX. Both the control and RDX-dosed rats were monitored for up to 3 hr after dosing.

### Measurements of *in vivo* acetylcholinesterase (AChE) and RDX concentrations

Rats were administered vehicle or RDX as described above. At the time of seizure onset, the rats were euthanized and samples of frontal lobe and blood were taken for measurements of AChE activity and content of RDX. We determined AChE activity in frontal cortex homogenates using the 96-well microplate method of Padilla et al. (1998). A 2% (wet wt/vol) homogenate was made in 0.1 M Na-phosphate buffer (pH 8.0) plus 1% Triton, using a 20-sec burst (on ice) of a Polytron AJ 10/35 (Brinkman Instruments, Rexdale, Ontario, Canada). Total cholinesterase [brain cholinesterase is 95% AChE (Padilla et al. 1998)] was determined at 37°C by measuring the change in absorbance at 412 nm over 5 in in a 96-well plate reader (Bio-Tek Synergy HT, Winooski, VT) using acetylcholine iodide as substrate and 5,5′-dithio-bis[2-nitrobenzoic acid] as the colorimetric indicator. We generated a glutathione sulfhydryl standard curve for conversion of absorbance units into nanomoles of sulfydryl groups. AChE enzymatic activity in the brain samples was then calculated as micromoles of substrate hydrolyzed per minute per gram wet weight (ww) (Padilla et al. 1998). Blood and brain samples were assayed for RDX content by the U.S. Army Public Health Command Directorate of Laboratory Sciences using gas chromatography with electron capture detection (GC-ECD) (Bishop et al. 2003).

### Receptor binding assays

Neurotransmitter receptor binding assays for RDX were performed by Ricerca Biosciences (Concord, OH). The specific assays and catalog numbers are listed in Table 1. Initially, RDX was tested at a single concentration of 33 μM. Routine screening for receptor affinity is usually done with a compound concentration of 10 μM (Ricerca Biosciences). To better ensure the probability of a “hit” with RDX, the initial test of RDX was performed using 33 μM, a half-log higher concentration. Subsequently, a complete dose response of RDX was tested in the [^35^S]-*t*-butylbicyclophosphoorothionate ([^35^S]-TBPS) convulsant site assay (catalog no. 226830) using picrotoxin as standard; this assay is based on the method of Maksay (1993). The maximal inhibitory concentrations (IC_50_) were determined by a nonlinear least-squares regression analysis using MathIQ (ID Business Solutions Ltd., Guildford, Surrey, UK). The inhibition constant (*K*_i_) values were calculated using the equation of Cheng and Prusoff (1973), the observed IC_50_ of the tested compound, the concentration of radioligand employed in the assay, and the historical values for the dissociation constant (*K*_D_) of the ligand (obtained experimentally at Ricerca Biosciences).

### *In vitro* extracellular and whole-cell patch-clamp recordings

Acute amygdala slices were prepared from male Sprague-Dawley rats (200–250 g body weight) as described previously (Aroniadou-Anderjaska et al. 2001; Braga et al. 2003). The artificial cerebrospinal fluid (ACSF) consisted of 125 mM NaCl, 2.5 mM KCl, 2.0 mM CaCl_2_, 1.0 mM MgCl_2_, 25 mM NaHCO_3_, 1.25 mM NaH_2_PO_4_, and 11 mM glucose, bubbled with 95% O_2_/5%CO_2_ to maintain a pH of 7.4. For extracellular field potential recordings, slices were transferred to an interface-type recording chamber maintained at 32°C, where they were perfused with ACSF at 0.7–1 mL/min. Spontaneous activity was recorded in the basolateral amygdala (BLA) in the gap-free mode (continuous recordings), while evoked field potentials were sampled by delivering single stimulation pulses to the external capsule at 30-sec intervals. Recording glass pipettes were filled with ACSF and had a resistance of approximately 5 MΩ. Bipolar stimulating electrodes were constructed from twisted stainless steel wire, 50 μm in diameter. Spontaneous and evoked analog signals were digitized using pClamp10 software (Molecular Devices, Division of MDS Analytical Technologies, Sunnyvale, CA).

For whole-cell recordings, slices were transferred to a submersion-type recording chamber (0.7 mL volume), where they were continuously perfused with oxygenated ACSF at a rate of 3–5 mL/min. The techniques employed were similar to those described previously (Braga et al. 2003; Pidoplichko and Dani 2005). All experiments were performed at 32–33°C. We visualized neurons under infrared light using Nomarski optics of an upright microscope (Zeiss Axioskop 2; Carl Zeiss MicroImaging, Thornwood, NY) equipped with a CCD-100 camera (Dage-MTI, Michigan City, IN). Tight-seal (> 1 GΩ) whole-cell recordings were obtained from the cell body of pyramidal-shaped neurons in the BLA region. Access resistance (5–24 MΩ) was regularly monitored during recordings, and cells were rejected if the resistance changed by > 15% during the experiment. Patch electrodes were fabricated from borosilicate glass and had a resistance from 3 to 4 MΩ when filled with solution A (60 mM CsCH_3_SO_3_, 60 mM KCH_3_SO_3_, 10 mM KCl, 10 mM EGTA, 10 mM HEPES, 5 mM Mg-ATP, 0.3 mM NaGTP, pH 7.2; 280–290 mOsm/kg) or with solution B (135 mM Cs-gluconate, 10 mM MgCl_2_, 0.1 mM CaCl_2_, 1 mM EGTA, 10 mM HEPES, 2 mM Na-ATP, 0.2 mM Na_3_GTP, pH 7.2; 280–290 mOsm/kg). Solution B (high Cs^+^) stabilizes cell leakage (allowing prolonged periods of recording) but depresses the hyperpolarization-activated current (*I*_h_), whereas solution A (lower Cs^+^) still keeps the leakage low without affecting the *I*_h_ current. Neurons were voltage-clamped at +40 mV (intrapipette solution A) or at −70 mV (intrapipette solution B) holding potential, using an Axopatch 200B amplifier (Axon Instruments, Foster City, CA).

We performed pressure application of GABA with the help of the push–pull experimental arrangement (Pidoplichko and Dani 2005). Pressure was applied to the pipette via a Picospritzer (General Valve Division, Parker Hannifin Corp., Fairfield, NJ), set at about 225 Pa (30 psi). A motorizer (Newport, Fountain Valley, CA) was coupled with the approach/withdrawal (push–pull) actuator of a micromanipulator (Burleigh PCS-5000 series; EXFO Photonic Solution Inc., Mississauga, Ontario, Canada). Motorizer movement and duration of application pulses were controlled with a Master-8 digital stimulator (AMPI; Jerusalem, Israel). Ionic currents were amplified and filtered (1 kHz) using an Axopatch 200B amplifier, with a four-pole low-pass Bessel filter, and were digitally sampled (up to 5 kHz). Currents were recorded using pClamp10 software (Axon Instruments) and further analyzed using OriginLab (Northampton, MA) and Mini60 software (Synaptosoft, Fort Lee, NJ).

We used the following receptor/channel antagonists during whole-cell recordings: 6-cyano-7-nitroquinoxaline-2,3-dione (CNQX), an α-amino-3-hydroxyl-5-methyl-4-isoxazole-propionate (AMPA)/kainate receptor antagonist; d-2-amino-phosphonovalerate (AP-5), an *N*-methyl-d-aspartic acid (NMDA) receptor antagonist; and SCH50911, a GABA_B_ receptor antagonist, all from Tocris Cookson (Ballwin, MO). We also used bicuculline methiodide, a GABA_A_ receptor antagonist, and tetrodotoxin, a sodium channel blocker, both from Sigma Chemical Co. (St. Louis, MO).

### Statistical analysis

Statistical significance was determined using unpaired or paired Student’s *t*-test. Differences were considered statistically significant when the probability for error was < 0.05. Data are presented as mean ± SE.

## Results

### RDX administration induces seizure activity *in vivo*

**Figure 1:**
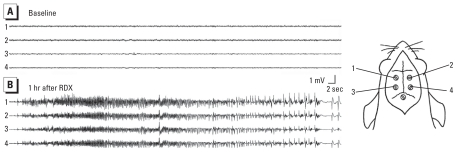
*In vivo* administration of RDX caused generalized electrographic seizures in rats. (*A*) Baseline EEG recording before RDX administration. (*B*) Representative EEG activity showing a generalized seizure, 1 hr after RDX administration (75 mg/kg, by gavage). Traces in *A* and *B* were recorded (top to bottom) from a left frontal (1), right frontal (2), left parietal (3), and right parietal (4) cortical screw electrode, as shown in the diagram.

We characterized RDX-induced seizures *in vivo* using behavioral observations and EEG recordings from surface-implanted electrodes. Rats (*n* = 4) were administered RDX (75 mg/kg) via oral gavage and allowed to move freely in their cages. Eleven to 16 min after RDX administration, rats presented one or more myoclonic twitches, followed by a single tonic-clonic seizure. The myoclonic twitch was accompanied, in the EEG, by a single sharp wave. We observed rhythmic, continuous, and high-amplitude polyspikes in the EEG during tonic-clonic seizures (Figure 1). This pattern of myoclonic twitches preceding a tonic-clonic seizure continued such that each rat had three or four tonic-clonic seizures within the next 1.5 hr before an episode of wild running/jumping. After the running/jumping, a final lasting tonic-clonic seizure with loss of righting reflex continued until death at 2–3 hr after RDX administration. Control rats that received only vehicle solution displayed normal behavior and normal electrographic activity.

### Effects of RDX on brain AChE

Organophosphorus pesticides and nerve agents are potent inhibitors of peripheral and central nervous system AChE. Exposure to these agents causes excessive salivation and lacrimation in addition to seizure induction (Shih et al. 2003). Because of anecdotal reports of increased salivation and/or lacrimation associated with RDX intoxication (Burdette et al. 1988; Crouse et al. 2006; Schneider et al. 1977), we speculated that RDX-induced seizures might involve a similar inhibition of brain AChE. To examine this possibility, we administered RDX (75 mg/kg) or vehicle solution to rats (*n* = 6/group). Seizures appeared 10–21 min after dosing, but we did not observe increased salivation or lacrimation. At the onset of seizures, the rats were euthanized, and samples of the frontal lobe and blood were collected for measurements of AChE activity and content of RDX. AChE activity in the vehicle- and RDX-treated groups was identical (6.6 ± 0.4 μmol/min/g ww). Thus, seizure induction by RDX does not involve inhibition of AChE.

### Blood and brain concentrations of RDX during seizures

**Figure 2:**
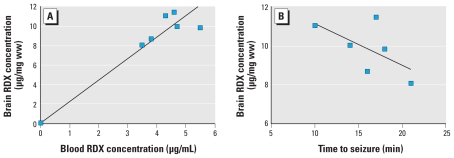
Bioavailability of RDX at the time of RDX-induced seizure onset. (*A*) Correlation between blood and brain RDX concentrations at seizure onset (CC = 0.81). (*B*) Correlation between RDX brain concentration and time to seizure onset; the negative correlation (CC = −0.61) indicates that the higher the brain concentration of RDX, the shorter the time to seizure initiation.

Analysis of the blood and frontal cortex samples taken at the onset of RDX-induced seizures indicated a direct correlation of blood to brain concentrations of RDX (Figure 2A); the correlation coefficient (CC) was 0.81. This result indicates that RDX readily enters the brain in direct proportion to the level of RDX in the blood after intestinal absorption. The correlation of brain RDX concentration with time to seizure onset after the oral gavage dose is presented in Figure 2B (CC = −0.61). These data indicate that the higher the brain concentration of RDX, the shorter the time interval between RDX administration and seizure onset.

### RDX binds to the convulsant site of the GABA_A_ receptor

**Figure 3:**
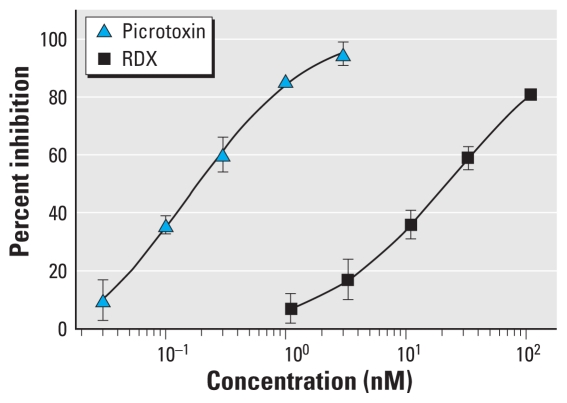
Dose–response curve for RDX and picrotoxin on [^35^S]-TBPS binding. The assays were performed in triplicate using a standard binding assay with rat brain membranes (Maksay 1993). The calculated *K*_i_ for RDX is 21.1 ± 2.1 μM.

To determine the binding sites of RDX in the brain that may be involved in seizure induction, we screened a battery of neurotransmitter receptors for affinity to RDX. The receptors assayed included those implicated as targets of known convulsants, such as the glutamate family of receptors, nicotinic and muscarinic acetylcholine receptors, the glycine receptor, the family of GABA_A_ receptor ligand sites, the batrachotoxin site of the sodium channel (site 2) (Catterall et al. 1981), and several others (Table 1). Out of this comprehensive list, the only binding site for which RDX had significant affinity was the TBPS/picrotoxin/*t*-butylbicycloorthobenzoate (TBOB) convulsant site of the GABA_A_ receptor (Kalueff 2007; Maksay 1993). RDX inhibited [^35^S]-TBPS and [^35^S]-TBOB binding by > 70% at the screening RDX concentration of 33 μM. In Figure 3, the full dose–response [^35^S]-TBPS binding curve is shown for both RDX and picrotoxin, which was used as a positive control; RDX has an apparent *K*_i_ of 21.1 ± 2.1 μM, compared with picrotoxin, which has an apparent *K*_i_ of 0.20 ± 0.042 μM.

### RDX reduces GABA_A_ currents and induces seizure-like activity in the BLA *in vitro*

Having found that RDX binds to the convulsant site of the GABA_A_ receptor, we next examined the functional consequences by determining the effect of RDX on GABA_A_ receptor– mediated currents in the basolateral nucleus of the amygdala (BLA). The amygdala plays a central role in seizure generation, with the BLA being the most important nucleus involved in this role (Aroniadou-Anderjaska et al. 2007, 2008; Mohapel et al. 1996; White and Price 1993), and previous studies have suggested that the amygdala is a target for RDX (Burdette et al. 1988; Levine et al. 1990; Macphail et al. 1985). In *in vitro* brain slices, we identified recorded neurons in the BLA as principal cells on the basis of their pyramidal shape and the presence of a current activated by hyperpolarization (*I*_h_ current). About 85% of amygdala neurons are principal pyramidal neurons displaying *I*_h_ (Park et al. 2007). We detected the presence of *I*_h_ by applying hyperpolarizing steps from the holding potential of −70 mV with 10-mV increments.

**Figure 4:**
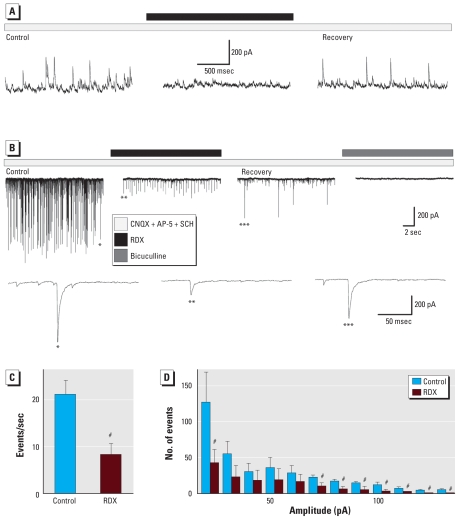
RDX reduced the frequency and amplitude of GABA_A_ receptor–mediated sIPSCs in the BLA; whole-cell recordings from principal neurons in the BLA were obtained in the presence of CNQX, AP-5, and SCH50911. (*A*) sIPSCs recorded at *V*_h_ = +40 mV. The outward currents were reduced in both frequency and amplitude by bath application of 30 μM RDX (black bar over the current trace) and recovered partially after 12-min washout of RDX. The recovered currents were completely blocked by the GABA_A_ receptor antagonist bicuculline (20 μM; data not shown). (*B*) sIPSCs recorded at *V*_h_ = −70 mV. Bath-applied RDX (30 μM) reduced both the frequency and the amplitude of the inward currents. After 12-min washout of RDX, sIPSCs recovered only partially. Bath-applied bicuculline (20 μM) blocked the spontaneous currents. The lower row illustrates example current traces at an expanded time scale from control recording (one asterisk), in RDX (two asterisks), and after washout of RDX (three asterisks). (*C*) Decrease in the frequency of high-amplitude events (the threshold was set at 20 pA) by RDX. (*D*) Decrease in amplitude of sIPSCs in the presence of RDX (bin width, 10 pA; *n* = 4 slices/cells). ^#^*p* < 0.05 by paired *t*-test.

First, we examined the effects of RDX on action-potential–dependent spontaneous inhibitory postsynaptic currents (sIPSCs) recorded at holding potential (*V*_h_) = +40 mV (Figure 4A) with intracellular/recording pipette solution A (see “Materials and Methods”) or at *V*_h_ = −70 mV (Figure 4B) with intracellular solution B, in the presence of CNQX (10 μM), AP-5 (50 μM), and SCH50911 (20 μM) to block AMPA/kainate, NMDA, and GABA_B_ receptors, respectively. Bath application of 30 μM RDX significantly reduced both the frequency and amplitude of the sIPSCs, from 21 ± 3 events/sec and 60 ± 10 pA (*n* = 4) in control conditions to 8 ± 2 events/sec and 31 ± 4 pA in the presence of RDX (*n* = 4, *p* < 0.05). Examples are shown in Figure 4A and B, and the group results are shown in Figure 4C and D. sIPSCs recovered only partially after 12-to 15-min washout of RDX. The recovered currents were completely blocked by the GABA_A_ receptor antagonist bicuculline (20 μM; Figure 4B).

**Figure 5:**
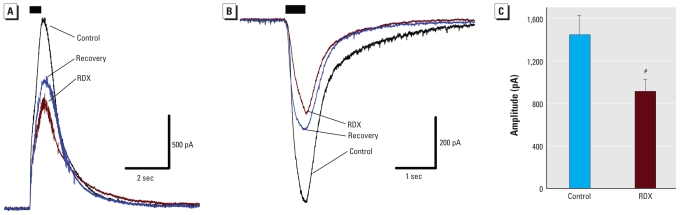
RDX reduced GABA-evoked currents in the BLA. Whole-cell recordings from principal neurons in the BLA were obtained in the presence of CNQX, AP-5, and SCH50911. (*A*) RDX (40 μM) reduced the outward postsynaptic current elicited by 500-msec pressure application of 200 μM GABA onto a pyramidal BLA neuron (*V*_h_ = +40 mV). (*B*) RDX (40 μM) reduced the inward postsynaptic current elicited by 500-msec pressure application of 200 μM GABA onto a pyramidal BLA neuron (*V*_h_ = −70 mV). In both *A* and *B*, each current trace is the average of four current recordings, and the black bar indicates the period of GABA application. After recording control current traces, RDX was applied to the bath; 4 min later, several GABA-evoked currents were recorded. Current amplitudes recovered partially after the washout of RDX. (*C*) Group data (mean ± SE; absolute current amplitude from both *V*_hold_ +40 mV and *V*_hold_ −70 mV experiments; *n* = 6) showing the effect of RDX on the amplitude of GABA-evoked currents. ^#^*p* < 0.05.

To directly demonstrate the blockade of GABA currents by RDX, we examined the effect of RDX on the currents induced by local pressure application of GABA. Pressure-applied GABA (200 μM) for 500 msec to pyramidal-shaped BLA neurons, in the presence of CNQX (10 μM), AP-5 (50 μM), and SCH50911 (20 μM), elicited currents that were significantly reduced by 40 μM RDX added to the bath (37 ± 3% reduction, mean ± SE; *n* = 6; Figure 5). Current amplitudes recovered only partially after the washout of RDX.

**Figure 6:**
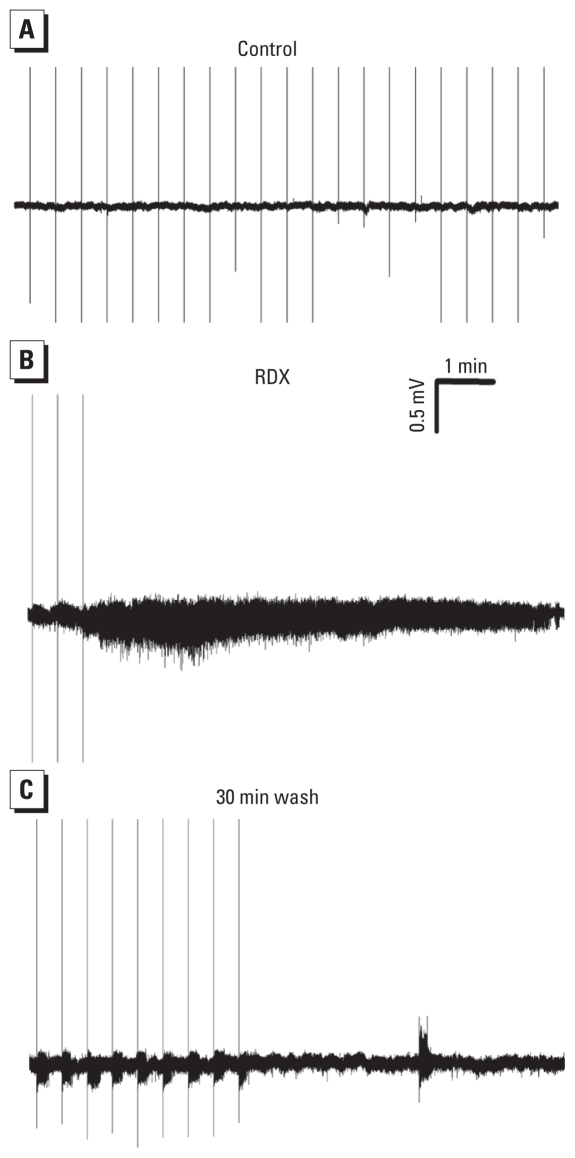
RDX induced seizure-like neuronal discharges in the BLA *in vitro*. Spontaneous field activity was recorded extracellularly from the BLA, in the gap-free mode, in amygdala slices. Single-pulse stimulation was applied at 30-sec intervals (regularly spaced vertical lines are the stimulus artifacts). (*A*) No spontaneous activity was present in control conditions (before application of RDX). (*B*) Bath application of RDX (100 μM) induced seizure-like discharges, which were triggered by the stimulus pulse, but were also present when stimulation was turned off. (*C*) The effects of RDX were not reversed after 30 min of wash.

To determine the impact of the reduction of sIPSCs by RDX on the overall activity of the BLA neuronal network, we applied RDX (100 μM) to the slice medium while recording extracellular field spontaneous activity. RDX induced prolonged, seizure-like neuronal discharges in the BLA within 15–25 min after exposure (*n* = 4; Figure 6). We applied single- pulse stimuli every 30 sec to sample the evoked field potential responses. Seizure-like activity was triggered by each stimulus pulse but was also present when stimulation was turned off (Figure 6B,C). The effect of RDX was not reversible at least after 40 min of RDX washout.

## Discussion

RDX has been used extensively as an explosive in both military and civilian applications. The health hazards from RDX exposure have been known since the 1940s, with brain seizures and status epilepticus as one of the major symptoms of RDX intoxication seen in humans (Barsotti and Crofti 1949; Stone et al. 1969) and rats (Burdette et al. 1988; Macphail et al. 1985; Schneider et al. 1978; Von Oettingen et al. 1949). However, until the present day there has been no information regarding the mechanisms by which RDX induces seizures. The major finding of the present study is that RDX binds to the picrotoxin convulsant site of the GABA_A_ receptor and inhibits GABA_A_ receptor–mediated synaptic transmission; this is likely to be the primary mechanism by which RDX induces brain seizures.

### RDX-induced seizures

Previous reports have described seizures in humans after accidental acute consumption of RDX (Hollander and Colbach 1969; Kasuske et al. 2009; Stone et al. 1969; Woody et al. 1986). Animal studies have also demonstrated the induction of seizures after RDX administration (Burdette et al. 1988; Crouse et al. 2006; Gust et al. 2009; Macphail et al. 1985; Martin and Hart 1974; Schneider et al. 1977; Von Oettingen et al. 1949). Because RDX causes rats to convulse within seconds after intravenous injection, Schneider et al. (1977) concluded that convulsions are caused by the parent compound rather than a neurotoxic metabolite of RDX. The present study supports this view because the brain concentration of RDX correlated positively with the time to seizure initiation and because RDX reduced GABAergic transmission and induced epileptiform activity in isolated brain slices. In addition, the present findings indicate that RDX is rapidly absorbed after oral administration and readily crosses the blood–brain barrier. A brain level of 8 μg/g ww was the lowest level we observed in the RDX-treated rats at the time of seizure onset, implying that this brain level is adequate for seizure initiation. The variability in blood/brain RDX concentrations among animals is probably related to differential absorption of the water-insoluble RDX in the methylcellulose suspension.

Burdette et al. (1988) suggested that the limbic system is significantly involved in RDX-induced seizures, based on the observation that amygdala kindling was accelerated in rats that received a relatively low daily dose of RDX. Here, we show that RDX reduces GABA_A_ receptor–mediated inhibitory synaptic transmission in the rat BLA, producing hyperexcitability and the appearance of seizure-like neuronal discharges *in vitro*. Consistent with the view that the amygdala is a primary target of RDX, and the well-known role of the amygdala in startle responses (Davis et al. 2008) and aggressive behavior (Mpakopoulou et al. 2008), rats that are administered RDX and do not reach the threshold for seizures display an increased acoustic startle response (Macphail et al. 1985), as well as an overall hyperreactivity and increased fighting (Levine et al. 1990).

### Mechanisms of RDX seizure induction

Many convulsants induce seizures by reducing GABAergic inhibition, either by competitively antagonizing GABA or by directly blocking chloride influx through the GABA_A_ channel (Kalueff 2007; Rowlett et al. 2005). The “cage convulsants,” compounds with cyclical structure, bind at a convulsant binding pocket inside the GABA_A_ ionophore at different—and possibly overlapping—positions. This “binding pocket” of the GABA_A_ channel is also referred to as the picrotoxin convulsant site (Ito and Ho 1994; Kalueff 2007; Maksay and van Rijn 1993). We found that RDX, a cyclical nitramine, binds to the GABA_A_ receptor convulsant site but does not bind to any of the other neurotransmitter or neuromodulator receptors we studied. RDX displaced TBPS, a noncompetitive (for GABA) ionophore blocker, which acts at the picrotoxin convulsant site (Ito and Ho 1994; Kalueff 2007; Maksay and van Rijn 1993). RDX also displaced TBOB, which appears to bind at the same site as TBPS, and with kinetics similar to those of TBPS (Maksay and van Rijn 1993). Thus, RDX binds inside the chloride ionophore at a convulsant site overlapping with TBPS. The potency of RDX at the GABA_A_ convulsant site (*K*_i_ of 21.1 μM) is similar to pentylenetetrazol, a convulsant commonly used in animal models of seizure and epilepsy that is also known to bind at the picrotoxin convulsant site (Coulter et al. 1990).

We tested the functional implications of RDX binding to the picrotoxin convulsant site in the amygdala, a limbic structure that plays a central role in the generation and propagation of seizures (Aroniadou-Anderjaska et al. 2008) and is a primary target for neurotoxins, including warfare neurotoxins such as nerve agents (Apland et al. 2009; Aroniadou-Anderjaska et al. 2009; Shih et al. 2003). We found that RDX significantly reduced the frequency and amplitude of action-potential–dependent sIPSCs in the BLA, the amygdala nucleus that plays the primary role in seizure generation (Aroniadou-Anderjaska et al. 2007, 2008; Mohapel et al. 1996; White and Price 1993). Consistent with the results from the binding studies described here, RDX reduced sIPSCs by a postsynaptic action on GABA_A_ receptors. This is supported by the significant reduction of the postsynaptic currents evoked by locally applied GABA in the presence of RDX. The prolonged, seizure-like neuronal discharges recorded in the BLA in the presence of RDX were probably a result of the reduced inhibitory tone (reduced sIPSCs) throughout the BLA network because of the blockade of the GABA_A_ channels by RDX. In a recent study in the northern bobwhite quail, Gust et al. (2009) found many molecular alterations in the brain after terminal RDX-induced seizures, including alterations in the expression of genes involved in the regulation of neuronal excitability.

RDX did not affect the activity of brain or peripheral blood AChE. The reported increased salivation and lacrimation after prolonged, low-dose RDX intoxication (Burdette et al. 1988; Crouse et al. 2006; Schneider et al. 1977) may be mediated by the effect of RDX at the GABA_A_ receptor, similar to the effects of type II pyrethroid esters (Ecobichon 2001).

## Conclusions

In the present study, we found that RDX induces seizures by binding to the picrotoxin convulsant site of the GABA_A_ ionophore, thereby reducing GABAergic inhibitory transmission. We studied the effect of RDX on GABAergic synaptic transmission in the basolateral nucleus of the amygdala. Along with previous studies (Burdette et al. 1988; Levine et al. 1990; Macphail et al. 1985), the present study suggests that the amygdala is involved in the seizurogenic effects of RDX; however, other brain regions may also be significantly involved. In addition, although we performed RDX binding studies for a number of neurotransmitter and neuromodulator receptors as well as sodium channels, the list certainly is not exhaustive; RDX might also bind to other channels that affect neuronal excitability, which we did not test in the present study. The mechanism of RDX seizure induction revealed in the present study in rats is probably similar in humans, because the binding characteristics of TBPS to the GABA_A_ receptor are similar in rat and human brain (Atack et al. 2007; Cole et al. 1984). Knowing that RDX induces seizures by binding to the GABA_A_ ionophore can guide treatment efforts in cases of RDX overexposure and contribute to the development of drugs that will prevent the onset of seizures without producing sedation or other undesirable effects. Furthermore, with other potential munitions in development by the military, knowledge of the molecular site and the mechanism of RDX action with respect to seizure induction can help prevent the development of explosives or other munitions that could pose similar health risks.

## Figures and Tables

**Table 1:** List of binding assays screened for affinity of RDX.

Catalog no.	Receptor/binding site	Percent inhibition
235010	Glutamate, nonspecific	7
232600	Glutamate, AMPA	−10
233000	Glutamate, NMDA, phencyclidine	2
258590	Nicotinic acetylcholine	9
254000	Muscarinic, nonselective, central	7
239000	Glycine, strychnine sensitive	2
279510	Sodium channel, site 2	16
228510	GABA, nonselective	4
226810	GABA_A_, chloride channel, TBOB	76^a^
226830	GABA_A_, chloride channel, TBPS	78^a^
226600	GABA_A_, flunitrazepam, central	5
226500	GABA_A_, muscimol, central	13
203500	Adrenergic α_1_, nonselective	−11
203900	Adrenergic α_2_, nonselective	−5
260500	Opiate, nonselective	7
226700	Peripheral benzodiazepine receptor	−6
268700	Purinergic P_2X_	10
271000	Serotonin 5-HT_1_, nonselective	26
271200	Serotonin 5-HT_1B_	−5
271910	Serotonin 5-HT_3_	−9
272000	Serotonin 5-HT_4_	12
279510	Sodium channel, site 2	−5
220320	Transporter, dopamine (DAT)	0
204410	Transporter, norepinephrine	−6
274030	Transporter, serotonin (SERT)	−5

Catalog numbers are from Ricerca Biosciences.

a Met criteria for significance.
