# **Erratum: Williams et al. “RDX Binds to the GABA_A_ Receptor–Convulsant Site and Blocks GABA_A_ Receptor–Mediated Currents in the Amygdala: A Mechanism for RDX-Induced Seizures” **[119:357–363 (2011)]

**DOI:** 10.1289/ehp.121-a146

**Published:** 2013-05-01

In Figure 2 of the paper by Williams et al. [Environ Health Perspect 119:357–363 (2011)], the units for “Brain RDX concentration” (*y*-axis) should have been micrograms per gram wet weight (µg/g ww) instead of micrograms per milligram wet weight (µg/mg ww). The corrected Figure 2 is shown below.

The authors regret the error.

**Figure 2:**
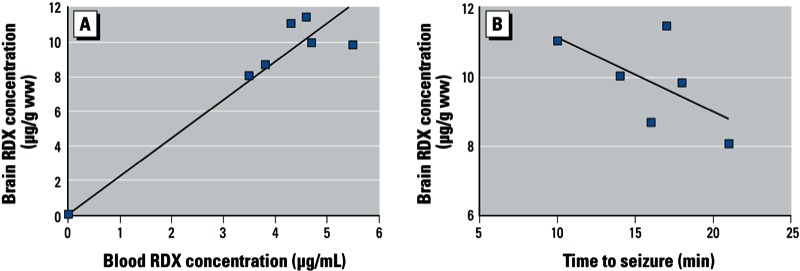
Bioavailability of RDX at the time of RDX-induced seizure onset. (*A*) Correlation between blood and brain RDX concentrations at seizure onset (CC = 0.81). (*B*) Correlation between RDX brain concentration and time to seizure onset; the negative correlation (CC = –0.61) indicates that the higher the brain concentration of RDX, the shorter the time to seizure initiation.

